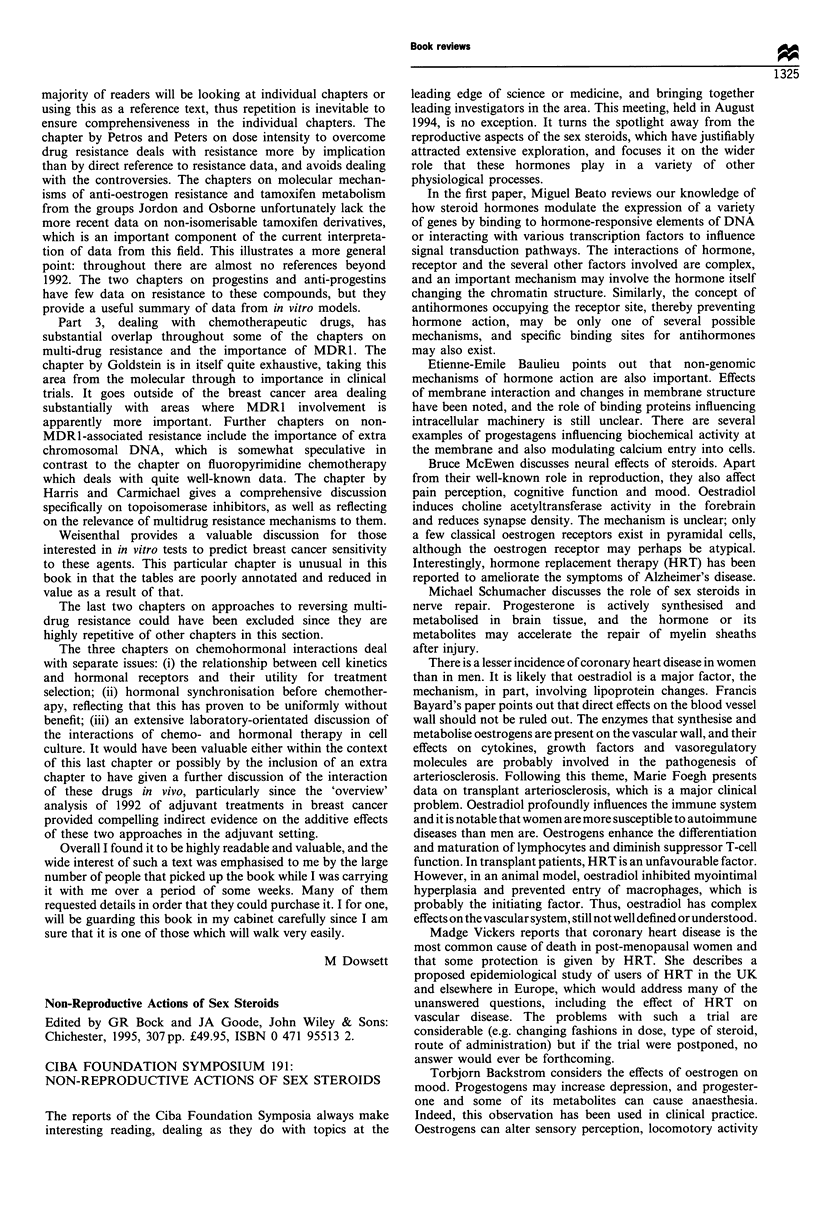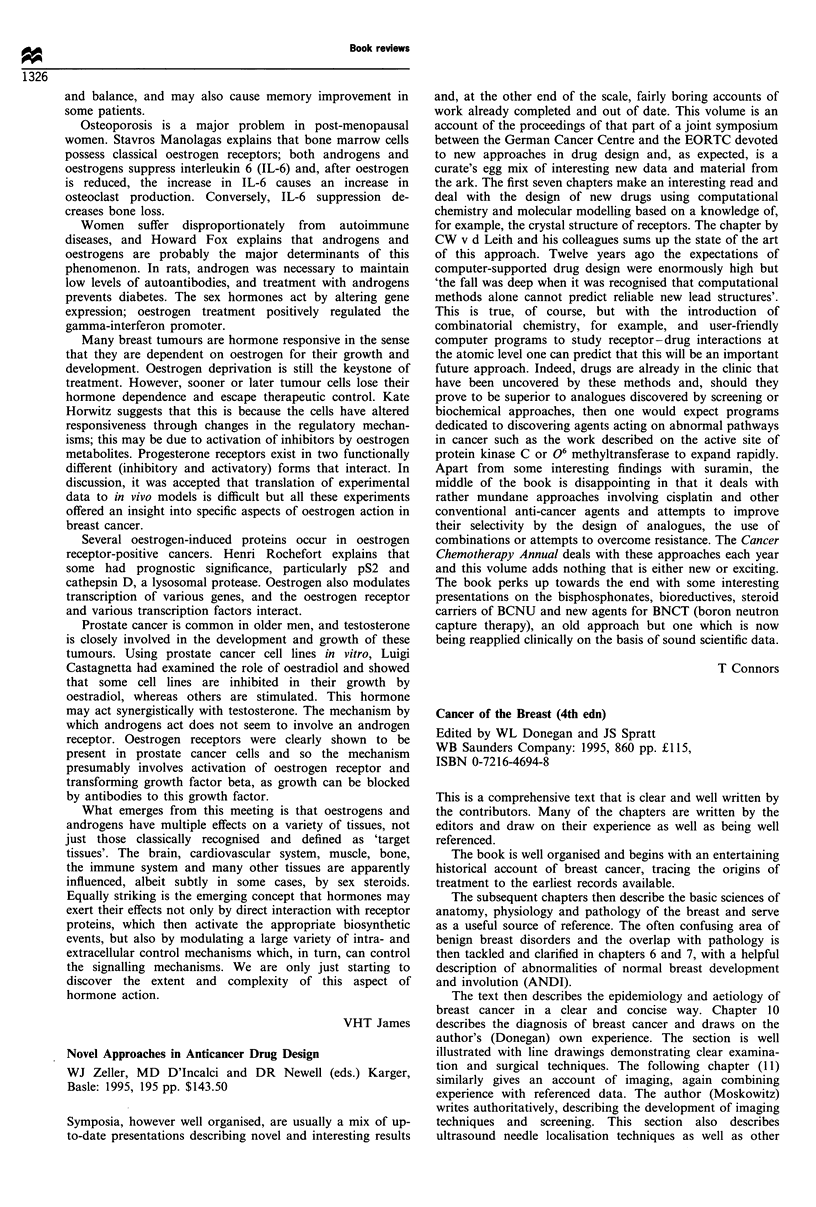# Non-reproductive actions of sex steroids

**Published:** 1996-10

**Authors:** V H T James


**Non-Reproductive Actions of Sex Steroids**

Edited by GR Bock and JA Goode, John Wiley & Sons: Chichester, 1995, 307 pp. £49.95, ISBN 0 471 95513 2.

CIBA FOUNDATION SYMPOSIUM 191:
NON-REPRODUCTIVE ACTIONS OF SEX STEROIDS

The reports of the Ciba Foundation Symposia always make interesting reading, dealing as they do with topics at the leading edge of science or medicine, and bringing together leading investigators in the area. This meeting, held in August 1994, is no exception. It turns the spotlight away from the reproductive aspects of the sex steroids, which have justifiably attracted extensive exploration, and focuses it on the wider role that these hormones play in a variety of other physiological processes.

In the first paper, Miguel Beato reviews our knowledge of how steroid hormones modulate the expression of a variety of genes by binding to hormone-responsive elements of DNA or interacting with various transcription factors to influence signal transduction pathways. The interactions of hormone, receptor and the several other factors involved are complex, and an important mechanism may involve the hormone itself changing the chromatin structure. Similarly, the concept of antihormones occupying the receptor site, thereby preventing hormone action, may be only one of several possible mechanisms, and specific binding sites for antihormones may also exist.

Etienne-Emile Baulieu points out that non-genomic mechanisms of hormone action are also important. Effects of membrane interaction and changes in membrane structure have been noted, and the role of binding proteins influencing intracellular machinery is still unclear. There are several examples of progestagens influencing biochemical activity at the membrane and also modulating calcium entry into cells.

Bruce McEwen discusses neural effects of steroids. Apart from their well-known role in reproduction, they also affect pain perception, cognitive function and mood. Oestradiol induces choline acetyltransferase activity in the forebrain and reduces synapse density. The mechanism is unclear; only a few classical oestrogen receptors exist in pyramidal cells, although the oestrogen receptor may perhaps be atypical. Interestingly, hormone replacement therapy (HRT) has been reported to ameliorate the symptoms of Alzheimer's disease.

Michael Schumacher discusses the role of sex steroids in nerve repair. Progesterone is actively synthesised and metabolised in brain tissue, and the hormone or its metabolites may accelerate the repair of myelin sheaths after injury.

There is a lesser incidence of coronary heart disease in women than in men. It is likely that oestradiol is a major factor, the mechanism, in part, involving lipoprotein changes. Francis Bayard's paper points out that direct effects on the blood vessel wall should not be ruled out. The enzymes that synthesise and metabolise oestrogens are present on the vascular wall, and their effects on cytokines, growth factors and vasoregulatory molecules are probably involved in the pathogenesis of arteriosclerosis. Following this theme, Marie Foegh presents data on transplant arteriosclerosis, which is a major clinical problem. Oestradiol profoundly influences the immune system and it is notable that women are more susceptible to autoimmune diseases than men are. Oestrogens enhance the differentiation and maturation of lymphocytes and diminish suppressor T-cell function. In transplant patients, HRT is an unfavourable factor. However, in an animal model, oestradiol inhibited myointimal hyperplasia and prevented entry of macrophages, which is probably the initiating factor. Thus, oestradiol has complex effects on the vascular system, still not well defined or understood.

Madge Vickers reports that coronary heart disease is the most common cause of death in post-menopausal women and that some protection is given by HRT. She describes a proposed epidemiological study of users of HRT in the UK and elsewhere in Europe, which would address many of the unanswered questions, including the effect of HRT on vascular disease. The problems with such a trial are considerable (e.g. changing fashions in dose, type of steroid, route of administration) but if the trial were postponed, no answer would ever be forthcoming.

Torbjorn Backstrom considers the effects of oestrogen on mood. Progestogens may increase depression, and progesterone and some of its metabolites can cause anaesthesia. Indeed, this observation has been used in clinical practice. Oestrogens can alter sensory perception, locomotory activity and balance, and may also cause memory improvement in some patients.

Osteoporosis is a major problem in post-menopausal women. Stavros Manolagas explains that bone marrow cells possess classical oestrogen receptors; both androgens and oestrogens suppress interleukin 6 (IL-6) and, after oestrogen is reduced, the increase in IL-6 causes an increase in osteoclast production. Conversely, IL-6 suppression decreases bone loss.

Women suffer disproportionately from autoimmune diseases, and Howard Fox explains that androgens and oestrogens are probably the major determinants of this phenomenon. In rats, androgen was necessary to maintain low levels of autoantibodies, and treatment with androgens prevents diabetes. The sex hormones act by altering gene expression; oestrogen treatment positively regulated the gamma-interferon promoter.

Many breast tumours are hormone responsive in the sense that they are dependent on oestrogen for their growth and development. Oestrogen deprivation is still the keystone of treatment. However, sooner or later tumour cells lose their hormone dependence and escape therapeutic control. Kate Horwitz suggests that this is because the cells have altered responsiveness through changes in the regulatory mechanisms; this may be due to activation of inhibitors by oestrogen metabolites. Progesterone receptors exist in two functionally different (inhibitory and activatory) forms that interact. In discussion, it was accepted that translation of experimental data to *in vivo* models is difficult but all these experiments offered an insight into specific aspects of oestrogen action in breast cancer.

Several oestrogen-induced proteins occur in oestrogen receptor-positive cancers. Henri Rochefort explains that some had prognostic significance, particularly pS2 and cathepsin D, a lysosomal protease. Oestrogen also modulates transcription of various genes, and the oestrogen receptor and various transcription factors interact.

Prostate cancer is common in older men, and testosterone is closely involved in the development and growth of these tumours. Using prostate cancer cell lines *in vitro*, Luigi Castagnetta had examined the role of oestradiol and showed that some cell lines are inhibited in their growth by oestradiol, whereas others are stimulated. This hormone may act synergistically with testosterone. The mechanism by which androgens act does not seem to involve an androgen receptor. Oestrogen receptors were clearly shown to be present in prostate cancer cells and so the mechanism presumably involves activation of oestrogen receptor and transforming growth factor beta, as growth can be blocked by antibodies to this growth factor.

What emerges from this meeting is that oestrogens and androgens have multiple effects on a variety of tissues, not just those classically recognised and defined as 'target tissues'. The brain, cardiovascular system, muscle, bone, the immune system and many other tissues are apparently influenced, albeit subtly in some cases, by sex steroids. Equally striking is the emerging concept that hormones may exert their effects not only by direct interaction with receptor proteins, which then activate the appropriate biosynthetic events, but also by modulating a large variety of intra- and extracellular control mechanisms which, in turn, can control the signalling mechanisms. We are only just starting to discover the extent and complexity of this aspect of hormone action.

VHT James